# Does osteoporosis affect the healing of subcapital humerus and distal radius fractures?

**DOI:** 10.1016/j.jor.2020.05.004

**Published:** 2020-05-06

**Authors:** E.A. Gorter, B.M. Gerretsen, P. Krijnen, N.M. Appelman-Dijkstra, I.B. Schipper

**Affiliations:** a Leiden University Medical Center, Departments of Trauma Surgery, Center for Bone Quality, Leiden, P.O. Box 9600, 2300, RC, Leiden, the Netherlands; b Leiden University Medical Center, Departments of Internal Medicine, Center for Bone Quality, Leiden, P.O. Box 9600, 2300, RC, Leiden, the Netherlands

**Keywords:** Osteoporosis, Fracture, Fracture healing, Delayed union, Non-union

## Abstract

**Introduction:**

Animal models indicate that osteoporosis may negatively influence the fracture healing process, but clinical studies on this topic are scarce. In this study we investigated the effect of osteoporosis on fracture healing in patients with an upper extremity fracture.

**Methods:**

This retrospective cohort study included all patients aged 50 years or older, with a fracture of the proximal humerus or the distal radius treated in the period June 2012 to July 2015 and a DEXA scan within a year after fracture. The incidence of delayed-union and non-union were compared between patients with or without osteoporosis (BMD T score ≤ −2.5SD). A secondary analysis was performed with a more pragmatically definition; BMD T score ≤ −2.5SD or a proximal humerus fracture with a T-score between −2.5SD and −1.0SD.

**Results:**

Osteoporosis was diagnosed in 133/455 patients (29.2%). A total of 461 fractures (distal radius n = 311 and proximal humerus n = 150) were treated. Radiological delayed- or non-union was described in 11/461 cases (2.4%); all proximal humerus fractures of which 6 cases (1.3%) were clinically manifest. The incidence of delayed- or non-union in fracture treatment did not differ between patients with osteoporosis (5/137 fractures) and the patients without osteoporosis (6/324 fractures) (p = 0.27). In the second analysis a significantly higher incidence was found in patients with osteoporosis (10/214 fractures vs 1/247 fractures p = 0.003)

**Conclusions:**

The results of this study suggest that osteoporosis does not significantly influence the progress of fracture healing in distal radius and proximal humerus fractures, although there seems to be a tendency towards a negative effect.

## 1 Introduction

Osteoporosis is a skeletal disorder that is characterised by low bone mass and micro-architectural deterioration of bone structure, resulting in bone fragility and an increased fracture risk.1 Remaining lifetime probability of a major osteoporotic fracture at the age of 50 is almost 50% for women and 22% for men.^2^ These fractures substantially contribute to excess morbidity, mortality and health care costs which will continue to rise the coming years.^2^, ^3^, ^4^ Fractures of the distal forearm and the proximal humerus are the most common osteoporotic fractures, besides vertebral and hip fractures.^2^^,^^3^

Mechanical and biological factors that are involved in the complex process of fracture healing seem to be affected negatively by osteoporosis.^1^^,^^5^ These effects of osteoporosis have mainly been studied in animal models of postmenopausal osteoporosis.^1^^,^^6^, ^7^, ^8^ Studies showed reduced bone mass and mechanical strength^8^ of the bone after completion of healing,^5^^,^^8^ and fracture healing appeared to be delayed^6^^,^^8^ with respect to callus mineralization. ^1^Also the progenitor cell recruitment, differentiation, and proliferation during the early phase of fracture healing were found to be impaired in the presence of postmenopausal osteoporosis, as were the angiogenesis and vasculogenesis during the early to mid-phase of fracture healing, the capacity of extracellular matrix production and callus formation during the mid-phase, and finally the capacity of callus remodelling in the later phase of fracture healing.^7^ Given these negative effects on the biomechanical processes of fracture healing, impaired fracture healing could be expected. However, clinical studies that describe the relation between osteoporosis and delayed fracture healing are however scarce and without consensus.^1^^,^^8^, ^9^, ^10^ Although the failure rate of implant fixation is increased in patients with osteoporosis,^1^^,^^8^ these patients had no clearly increased risk of delayed union or non-union.^8^ On the other hand, Nikolaou et al.^10^ found an obvious negative effect of osteoporosis on fracture healing time, whereas Wunnick et al.^9^ did not identify bone mineral density as a risk factor for non-union.

Interestingly, more clinical evidence is available on the effect of osteoporosis treatment on fracture healing. Treatment of osteoporosis with bisphosphonates, the most commonly used drugs,^3^ does not seem to delay fracture healing,^11^, ^12^, ^13^ although one systematic review concluded that it significantly prolongs time to union of distal radius fractures.^14^ Denosumab, another anti-resorptive drug, does not seem to delay fracture healing either.^13^ The bone stimulating drug Teriparatide, a recombinant parathyroid hormone analogue, appears to have a positive effect on fracture healing time.^12^^,^^13^

Because of the high incidence of osteoporotic fractures, there is a need for clinical studies elucidating the effect of osteoporosis itself on fracture healing. The aim of this exploratory study was to investigate if osteoporosis is associated with more fracture non-unions in patients with a proximal humerus or distal radius fracture compared to patients without osteoporosis.

## 2 Methods

### 2.1 Study design and patients

All patients of 50 years or older with a fracture of the proximal humerus or distal radius treated in the period June 2012 to July 2015 at the Leiden University Medical Center in Leiden, the Netherlands, were eligible for inclusion in this retrospective cohort study. According to the national protocol, all patients older than 50 years with a fracture were offered an osteoporosis screening, including a Dual Energy X-ray Absorptiometry (DEXA) scan and blood tests.

Patients were included if a DEXA-scan had been made within a year after the fracture. Patients with severe injuries (injury severity score ≥16), patients using osteoporosis treatment at the time of the fracture, and patients who went elsewhere for treatment/follow-up or were lost to follow-up, were excluded from the analysis. Patient characteristics (including age, gender, body mass index [BMI], medical history, smoking, alcohol consumption and use of medication) and fracture characteristics (including mechanism of trauma, AO fracture classification, open/closed fracture, treatment) were recorded from the medical files. The study was approved by the institutional Medical Ethics Review Board (protocol no. G17.034).

### 2.2 Osteoporosis and treatment

The epidemiologic definition of osteoporosis was based on the standardized (T) scores used in the WHO criteria that define osteoporosis as a BMD T score ≤ −2.5 SD.^2^ A secondary analysis was performed in which osteoporosis was defined as a BMD T score ≤ −2.5SD or between −2.5SD and −1.0SD in combination with a proximal humerus fracture.^15^ Also vitamin D (serum VitD25(OH)), calcium and Parathormone were measured. The endocrinologist analysed the results of the DEXA scan and blood parameters, and initiated subsequent treatment in case of osteoporosis or osteopenia according to national protocol,^15^ as well as screening for secondary osteoporosis. This information was recorded from the medical files. All patients were started on vitamin D supplementation (800IE daily) according to the national guideline, if they did not have this already. In case of a vitamin D deficiency (serum VitD25(OH) level <50 nmol/L), the dose was raised or patients were switched to monthly preparations. Calcium supplements were not started if dietary calcium intake was sufficient according to a standard dietary questionnaire since calcium intake in the Netherlands is high.

### 2.3 Outcome

The primary outcome measure of this study was the occurrence of delayed or non-union. Fracture healing was considered delayed or a non-union (1) in case of incomplete union/consolidation (i.e., incomplete bone bridging between fracture fragments on at least two cortical sides of the fracture) on a follow up X-ray 6 months or longer after trauma, or (2) if delayed or non-union was described as such in the patient files, or (3) if patients had received secondary surgery for delayed or non-union. Laboratory results and data on clinical and radiological fracture healing were recorded from the electronic hospital records. Follow-up radiographs were reviewed by a radiologist and one of the investigators separately. If no consensus existed, a third senior author was asked to review the radiographs and consensus was reached by discussion.

Secondary outcomes were complications registered in the patient records during the follow-up, including posttraumatic dystrophy, malunion (i.e.,consolidation in a nonanatomic position), neuropraxia, persistently decreased function and re-fracture.

### 2.4 Statistical analysis

The outcome parameters were compared between patients with osteoporosis, osteopenia and normal bone density. Patient characteristics are presented as mean and standard deviation (SD) or as number (%). Categorical variables were compared between patient groups using the Chi-square test, and continuous data were analysed using one-way analysis of variance (ANOVA), respectively. P-values <0.05 were considered statistically significant. Statistical analysis was performed with IBM SPSS Statistics for Windows, version 23 (IBM Corp., Armonk, N.Y., USA).

## 3 Results

### 3.1 Patients characteristics

During the study period, 481 patients met the inclusion criteria. Two patients had an injury severity score ≥16, 13 patients were treated for osteoporosis at the time of accident, ten received treatment/follow-up elsewhere and one patient was lost to follow-up. These patients were excluded from the analysis. The remaining 455 patients had a mean age of 68.2 years (SD 10.1, range 50–94) and were mostly women (85.7%) (Table 1).

**Table 1:** Characteristics of 455 patients.

	BMD T ≤ −2.5 SD		
	Osteoporosis (n = 133)	No osteoporosis (n = 322)	p-value
**General characteristics**			
Female gender, n (%)	121 (91%)	269 (83.5)	0.04
Age [years], mean (SD)	72.2 (10.0)	66.5 (9.7)	P < 0.001
BMI (n = 409), mean (SD)			
-BMI <18.5, n (%)	10 (8.1)	1 (0.4)	P < 0.001
-BMI 18.5–25, n (%)	59 (47.6)	95 (34.7)	
-BMI >25, n (%)	55 (44.4)	178 (65.0)	
Previous fractures, n (%)	71 (53.4)	159 (49.4)	0.44
Medical history, n (%)			
- Renal insufficiency	19 (14.3)	51 (15.9)	0.67
- Diabetes	11 (8.3)	24 (7.5)	0.77
- Hyper(para)thyroidism	9 (6.8)	24 (7.5)	0.79
- COPD	8 (6.0)	16 (5.0)	0.66
- Inflammatory bowel disease	1 (0.8)	1 (0.3)	0.54
- Liver function impairment	0 (0.0)	1 (0.3)	0.40
Two or more causes of secondary osteoporosis	6 (4.5)	20 (6.2)	0.48

**Use of medication**			
Vitamin D, n (%)	34 (25.6)	46 (14.3)	0.004
Corticosteroids, n (%)	22 (16.5)	34 (10.6)	0.08
NSAIDs, n (%)	7 (5.3)	20 (6.2)	0.70

**Intoxication (n=440)**			
Smoking, n= (%)	18 (13.8)	47 (15.2)	0.72
Alcohol, n (%)	63 (48.5)	186 (60.0)	0.03

**Endocrine characteristics, (n=448)**			
Calcidiol, mean (SD)	61.6 (31.6)	58.0 (29.9)	0.25
Vitamin D deficiency, n (%)	49 (36.8)	141 (44.8)	0.21

BMI: body mass index; COPD: chronic obstructive pulmonary disease; NSAID: nonsteroidal anti-inflammatory drug.

Osteoporosis (T < −2.5SD) was diagnosed in 133 patients (29,2%). On average, the DEXA scan was made after an mean of 7.4 weeks (SD 5.6; range 0–38). The results of the DEXA scan did not differ between the group of patients in which the scan was performed within 8 weeks compared to the group with a scan made after 8 weeks (p = 0.07). Fifty-one percent had sustained a previous fracture and 33.3% had a medical history that could cause a secondary osteoporosis (chronic obstructive pulmonary disease, inflammatory bowel disease, hyper(para)thyroidism, rheumatoid arthritis, diabetes, renal insufficiency or liver impairment). Eighty patients (17.6%) used vitamin D supplements at the time of the fracture, 27 (5.9%) patients used nonsteroidal anti-inflammatory drugs and 56 (12.3%) used corticosteroids. Patients with osteoporosis were more frequently female, older and had a lower BMI and used more frequent vitamin D supplementation and alcohol compared to the patients with no osteoporosis (Table 1).

A total of 179 patients were started on treatment with bisphosphonates or Denosumab. The majority of patients (n = 155; 86.6%) started with bisphosphonates according to the national guideline for osteoporosis in the Netherlands, and 24 patients were started on Denosumab.

The mean patient serum concentration of vitamin D was 59.0 nmol/L (SD 30.5, range 7.9–165) in 448 patients (unknown in 7 patients). Of these, 129 patients (28.8) had a sufficient vitamin D level (VitD25(OH) level >75 nmol/L), also 129 (28.8%) patients had insufficient levels (VitD25(OH) level 50–75 nmol/L) and 190 (42.4) had a vitamin D deficiency (VitD25(OH) level <50 nmol/L) of whom 58 (12.9%) had a severe vitamin D deficiency (VitD25(OH) level < 25 nmol/L).

### 3.2 Fracture characteristics and treatment

The 455 patients were treated for 461 fractures, 311 (67.5%) distal radius fractures and 150 proximal humerus fractures. The fracture characteristics are presented in Table 2. The fracture location and type were not related to the presence of osteoporosis (p = 0.75). The initial treatment was mostly non-operative for both fracture locations, 143/150 (95.3%) in case of proximal humerus fractures and 286/311 (92.0%) in case of distal radius fractures.

**Table 2:** Characteristics of 461 fractures, by bone density group.

	BMD T ≤ −2.5 SD		
	Osteoporosis (n = 137)	No Osteoporosis (n = 324)	p-value
**Fracture characteristics, n (%)**			
Intra articular distal radius fracture	60 (43.8)	151 (46.6)	0.75
Extra articular distal radius fracture	28 (20.4)	72 (22.2)	
Intra articular proximal humerus fracture	4 (2.9)	11 (3.4)	
Extra articular proximal humerus fracture	45 (32.8)	90 (27.8)	

High energy trauma, n (%)	1 (0.7)	3 (0.9)	0.83
Open fracture, n (%)	3 (2.2)	3 (0.9)	0.30
One or more additional fractures, n (%)	14 (10.2)	21 (6.5)	0.17
Conservative treatment, n (%)	128 (93.4)	301 (92.9)	0.84

### 3.3 Delayed- and non-union

The mean follow-up was 17 weeks (SD 15.1). Most of the patients were discharged from follow-up within 8 weeks (30.6%), 12 weeks (51,2%), 16 weeks (67%) or 6 months (82%), without signs of delayed or non-union. In 32 of the remaining 82 patients (39%), radiological follow-up was performed after 6 months and showed incomplete union in 11 cases. So in 11/461 (2.4%) fractures a radiologically delayed or non-union was recorded. Ten of the 11 non-united fractures had initially been treated conservatively and 1 had been treated operatively. The incidence of non-union did not differ significantly between the conservatively treated group and the operated patients (p = 0.78). In six of these cases (five initially treated conservatively and one operatively) a clinically delayed- or non-union was described as well, which resulted in secondary surgery in three cases and an expectative management resulting in an acceptable clinical situation in the other three patients. In the other five patients with a radiological delayed or non-union recorded this was not clinically manifest. The incidence of delayed- or non-union was not significantly higher in the group of patients with osteoporosis (DEXA T < -2.5SD) (5/137 fractures = 3.6%) compared to the patients with no osteoporosis (6/324 fractures = 1.9%) (p = 0.27) (Table 3). No significant difference in the incidence of delayed or non-union was found for age (p = 0.36), previous fractures (p = 0.33), presence of two or more secondary causes of osteoporosis in the medical history (p = 0.40), use of medication (p = 0.98) or vitamin D status (p = 0.85). In the secondary analysis (with osteoporosis defined as DEXA T < 2,5SD and patients with a proximal humerus fracture with a DEXA between −2.5 and −1.0), the incidence of delayed or non-union was significantly higher in the group of patients with osteoporosis (10/214 fractures = 4.7%) compared to the patients without osteoporosis (1/247 fractures = 0.4%) (p = 0.003). Since all 11 non-united fractures concerned patients with a proximal humerus fracture, this subgroup was evaluated separately. No difference in incidence of delayed or non-union was found between the conservatively treated (10/143 fractures = 7.0%) and operatively treated (1/7 fractures = 14.2%) (p = 0.42). Also the incidence of delayed- or non-union did not differ between the patients with osteoporosis (5/49 fractures = 10.2%) and no osteoporosis (6/101fractures = 5.9%) (p = 0.36). No significant difference in incidence of delayed or non-union was found for age (p = 0.28), previous fractures (p = 0.26), presence of two or more secondary causes of osteoporosis in the medical history (p = 0.26), use of medication (p = 0.79) or vitamin D status (p = 0.86). In the sub analysis, the incidence of delayed- or non-union was not significantly higher in the group of patients with osteoporosis (10/125 fractures = 8.0%) compared to the patients without osteoporosis (1/25 fractures = 4.0%) (p = 0.45).

**Table 3:** Outcome fracture union.

All fractures (n = 461)	Fracture union (n = 450)	Delayed or non-union (n = 11)	p-value
**Osteoporosis** (T-score < −2.5SD), n (%)	132 (29.3)	5 (45.5)	0.27
**Osteoporosis** (T score ≤ −2.5 SD or between −2.5SD and −1.0SD in combination with a proximal humerus fracture), n (%)	204 (45.3)	10 (90.9)	0.003

**Subcapital humerus fractures (n=150)**	Fracture union (n = 139)	Delayed or non-union (n = 11)	p-value
**Osteoporosis** (T-score < −2.5SD), n (%)	44 (31.7)	5 (45.5)	0.36
**Osteoporosis** (T score ≤ −1.0SD), n (%)	115 (82.7)	10 (90.9)	0.45

### 3.4 Other complications

In 35 of the 429 conservatively treated fractures (8.1%), one or more complications (other than delayed- or non-union) during treatment were reported: persistent functional impairment in 25 cases (5.8%), mal-union in eight cases (1.9%) and re-fracture in two cases (0.4%). Sixteen fractures (3.7%) with initially non-operative treatment were operated secondarily, mostly due to deterioration of fracture alignment (13 cases) and in three cases due to delayed or non-union as described earlier. In 9 of the 32 surgically treated fractures (28.1%) a complication other than delayed- or non-union occurred during follow-up, including persistent functional impairment in seven cases (21.9%) and neuropraxia of the median nerve in two cases (6.2%). The complication rate was not significantly higher in the group of patients with osteoporosis compared to the patients without osteoporosis (p = 0.40), secondary analysis (p = 0.07).

## 4 Discussion

This study retrospectively investigated the effect of osteoporosis on fracture healing in a 455 patients of 50 years and older with 461 proximal humerus or distal radius fractures. Osteoporosis was prevalent in 29.2% of the patients. Radiological delayed or non-union was described 11 cases (2.4%), all proximal humerus fractures of which 6 cases (1.3%) were clinically manifest. The presence of osteoporosis did not seem to influence the incidence of delayed- or non-union, nor the incidence of other complications, although there seems to be a tendency towards a negative effect.

In this study, osteoporosis was primarily defined according to the WHO DXA criteria.^2^ Intervention BMD thresholds have ranged from Tscores of −3 SD to −1.5 SD depending on the clinical context, the country or on health economic factors.^2^ Therefore we performed a secondary analysis with the definition/threshold of osteoporosis as it is used in our National guideline.^15^

In general, the incidence of non-union is mainly investigated in diaphyseal fractures and described to be around 10%.^16^^,^^17^ In our study we found a relatively low number of delayed- and non-unions. Analysing specifically the group of proximal humerus fractures, the incidence was 7.3%. In literature there is no consensus about the time frame (3–6 months after injury) on the definition of non-union in case of proximal humerus fractures.^18^^,^^19^ With a median time of 13 weeks to achieve fracture union or bridging callus in case of a non-surgically treated fracture,^19^ a period of 6 months were chosen as time cut-off point. Papakonstantinou et al.^18^ found delayed- and non-union in up to 32.4% and 8.2% of cases respectively, in patients with proximal humerus fractures. The reason that their numbers are higher compared to our findings might be the shorter time frame in their definition of delayed and non-union (delayed union >60 days, non-union >90 days) and the inclusion of more displaced and intra-articular fractures in their study. In the review of Cadet et al.^19^ an incidence of non-union was described between 1.1% and 10% in proximal humerus fractures, depending on dislocation and the presence of multiple fracture parts. We found no radiological delayed- and non union in distal radius fractures which seems to be in accordance with the literature, which states that it is quite rare.^20^

Clinical studies evaluating delayed bone healing in association with osteoporosis are scarce. The finding in our study that osteoporosis does not seem to influence the incidence of delayed-non-union is supported by the study of Wunnik et al.^9^ They found that in fracture patients around 70 years of age, bone mineral density was not a risk factor for non-union (based on a X-ray 6 months after injury) in non-typical fragility fracture locations. Nikolaou et al.^10^ found prolonged fracture healing time in patients >65 years (mean age 76.8 years) with an osteoporotic femur shaft fracture (delayed union >24 weeks and pseudarthrosis >32 weeks). However, they compared this patient group with a group of patients aged between 18 and 40 years old, (mean age 25.3 years), with the addition that the presence of risk factors for delayed union did not differ between the groups. Compared to these other studies,^9^^,^^10^ our study results are based on a general outpatient population with typical fragility fractures.

Regarding the effect of osteoporosis treatment on fracture healing, our study did not have sufficient statistical power to evaluate the influence of osteoporosis treatment on fracture healing. Another reason not to evaluate this relation was that the effect of osteoporosis treatment might have been diminished due to the time span between the accident and the start of osteoporosis therapy, which was on average 7 weeks until the DEXA-scan plus the additional time associated with the referral to the endocrinologist. This could be considered as a limitation of the study, although studies by Li et al.,^11^ Silverman et al.^12^ and Hegde et al.^13^ suggested no influence of treatment with bisphosphonates on fracture healing, while Molvik et al.^14^ concluded that time to union was significantly prolonged in patients treated with bisphosphonates compared to those who did not receive bisphosphonates, especially in distal radius fractures. Hegde et al.^13^ postulated that Denosumab does not delay fracture healing. The main limitation of our study was the relatively low number of patients with delayed or non-union, which resulted in a risk of a type 2 error and did not allow a multivariable analysis in order to correct for potential confounders. The primary analysis with the definition of osteoporosis (BMD T score < 2.5SD) and both the analyses in the proximal humerus fracture subgroup showed a non-significant trend towards more delayed- and non-unions in the osteoporotic group, though the post hoc power analysis showed insufficient power. However, analyzing osteoporosis from a clinical point of view (BMD T score < 2.5SD and patients with a BMD T score between −1.0SD and −2.5 who suffered a proximal humerus fracture) significantly (p = 0.003) more delayed- and non-unions were found in the osteoporosis group, with a post hoc power of 0.82.

The study further holds all limitations related to a retrospective study design. Data collection was based on the chart notes by different physicians, which may have led to information bias. Clinical union of fractures was determined based on the patient records, without a standardized functional fracture healing test or CT scans. Regarding the registration of clinical complications, we assumed that already discharged patients would have returned after follow up if they encountered complications. It is however possible that patients went to another hospital for additional treatment. Radiographic follow-up was not standardized, so the incidence of radiological delayed union could not be determined in all the patients who had a follow-up visit after 6 months. Also the diagnosis delayed union was based on plain films and not on CT-scan, which would be more accurate. We do appreciate the insecurities that arise from judging radiographs for fracture union or non-union.^21^^,^^22^ This may have resulted in an underestimation of the extent of fracture union, and could help explain the finding that in ten patients with a radiological delayed- or non-union only six were clinically manifest. Although in general practice, only clinical delayed- and non-unions will be treated.

In conclusion, the results of this study suggest that osteoporosis does not significantly influence the progress of fracture healing, although there seems to be a tendency towards a negative effect. Considering the actual and expected prevalence of osteoporosis in patients older than 50 years and the potential clinical implications of the effect of osteoporosis (treatment) on fracture healing, prospective research is needed to confirm this study's findings. Additionally, the effects of osteoporosis treatment on bone healing should be further addressed.

## Author contributions

EG: Conceptualization, Methodology, Writing - Original Draft.

BG: Data Curation, Formal analysis.

PK: Writing - Review & Editing; Project administration.

NA: Conceptualization, Writing - Review & Editing.

IS: Conceptualization; Writing - Review & Editing, Supervision.

## Declaration of competing interest

None of the authors have any conflict of interest to declare.
